# Costs of Providing Intensive Care for Adult Non-survivors in a Caribbean Teaching Hospital

**DOI:** 10.7759/cureus.12141

**Published:** 2020-12-18

**Authors:** Venkata Gosula, Seetharaman Hariharan

**Affiliations:** 1 Anaesthesia and Intensive Care, Eric Williams Medical Sciences Complex, Trinidad, TTO; 2 Anaesthesia and Intensive Care, The University of the West Indies - St. Augustine, St. Augustine, TTO

**Keywords:** icu costs, caribbean, non-survivors, activity-based costing

## Abstract

Introduction

Intensive Care Unit (ICU) is a resource intense area consuming a vast majority of the hospital’s budget. This study aimed to determine the costs of providing critical care to non-survivors in an adult ICU at a tertiary care teaching hospital in the Caribbean.

Methods

A chart review of non-survivors over a period of nine months was done in an adult ICU. Admission diagnoses, Simplified Acute Physiology Score (SAPS II) score, daily laboratory investigations, drugs, and all therapeutic interventions including mechanical ventilation were recorded. Activity-based costs were prospectively estimated by data obtained from ICU flowsheets, nursing-activity scores, and various hospital departments.

Results

A total of 316 days of ICU intervention data were collected from the 39 non-survivors enrolled. The median patient age was 56 years. The median ICU length of stay (LOS) and the median duration of mechanical ventilation were five days. The median SAPS II score was 62. One-third of patients had cardiovascular problems and 28% were surgical patients. The total cost of providing ICU care for the non-survivors was US$ 765,233 with an average cost of US$ 19,621 per patient. Human resources (39%) and consumables (29%) were the highest components of costs. Patients who had a cardiac arrest before admission consumed more resources. A higher SAPS II score predicted a shorter LOS (p=0.01) and lower costs (p=0.03).

Conclusions

ICU care for non-survivors consume significantly high resources. Stringent admission protocols and consideration of medical futility at an earlier stage, using prognostic models and clinical criteria may prevent unnecessary interventions and costs.

## Introduction

Intensive care is known to be expensive in most countries, consuming disproportionately large portions of budgetary resources for a smaller number of patients [1]. As early as 1990, the Intensive Care Unit (ICU) costs were estimated to comprise about 20% of the total hospital expenditure in the USA [2]. Within a decade from 2000 to 2010, there has been a reported increase of 92% in the costs for intensive care in the USA alone, increasing from 56 to 108 billion US dollars [3]. In 2015, the total healthcare budget for Trinidad & Tobago was 7% of the total annual budget [4]. The annual expenditure incurred for an ICU at one of the teaching hospitals in Trinidad was found to be US$ 3,216,226 in 2012, which was 3.64% of the total hospital expenditure [Brochure; Health Economics Unit, UWI St. Augustine, Costing of Health services of Trinidad & Tobago; 2012].

Worldwide there is increasing pressure to improve ICU performance and quality of care in ways that will reduce costs [5]. Most critically ill patients admitted to an ICU need special therapeutic interventions, which are evolving rapidly in terms of technology, therefore leading to an increase in the associated costs. Resource consumption varies widely between individual units as well as patients and is influenced by multifarious factors including case-mix, illness severity, length of stay, and extent of therapeutic interventions, thereby making these parameters ideal for cost estimation [6]. Cost estimation helps in not only allocating resources efficiently in every setting but also contributes to devising measures for quality improvement in other similar settings.

Estimating ICU costs is a complicated task and data collection for costs is quite cumbersome. A number of studies have been conducted worldwide, using different approaches for determining the ICU costs. The cost per patient admission in ICU widely varies in accordance with the setting, ranging between US$ 1783 to US$ 78,435 [6]. Most of these variations are attributed to a number of different factors like advances in healthcare technology and its application, ICU organization structure e.g., differing staff/patient ratio, treatment options influencing the patient selection and costs, but most importantly the varying costing methodologies used in different studies [7].

Although, the total ICU costs may provide a rough estimate of the resource consumption in ICU as a proportion of the total hospital budget, specific data on the cost of ICU care e.g., for survivors versus non-survivors, and the association of costs with the severity of illnesses of patients may be important, especially to implement quality improvement measures such as ICU admission protocols. The mean cost of ICU care per patient in Trinidad & Tobago was US$ 5228 as reported in a previous study published in 2007 [8]; however, there was no comparison of costs between survivors and non-survivors. A study done in Barbados showed the cost of ICU admission to be US$ 6818, which also compared the costs for survivors (US$ 6134) to non-survivors (US$ 10445) [9]. Intuitively, the cost of ICU admission for non-survivors should be significantly higher than survivors, which may correlate with the severity of illness and the intense amount of therapeutic interventions needed for the sickest of the patients. Hence, there is a need of studying the details of costs and resource consumption in non-survivors to get a better picture of the value of these interventions. In survivors, because the patient’s life is saved, the cost incurred in providing critical care support may be arguably justifiable. However, in non-survivors, it may be worthwhile to study the pattern of costs and resource consumption, their clinical severity of illness at admission, and the possible factors that might help in minimizing the wastage of costs. Prolonging the length of stay in ICU with organ support that eventually may not lead to patient survival is a clear example of wastage of resources both in fee-for-service (for the patient and the family) as well as free ICU services (for the exchequer) as in our setting.

Futile treatment in ICU or what is referred to as ‘inappropriate’ care is a controversial subject and has stirred a lot of debate in the literature [10]. The concept of futility in intensive care is yet to be widely accepted in developing countries like Trinidad & Tobago, although the ICU care is comparable to that in more developed countries [11]. The lack of standard definitions for futility and non-beneficial care leads to the perpetuation of aggressive ICU interventions and continuation of non-beneficial treatment, thereby leading to unnecessary prolongation of patient’s suffering without long-term benefits [12]. In 2013, the opportunity cost of futile treatment in ICU was estimated at US$ 2.6 million [13]. Missed opportunities were described in terms of delayed admissions from emergency departments, due to the ongoing futile treatment in the ICU [14]. However, although the cost of ICU care is involved in the equation, when rationing of ICU care becomes necessary, the process of decision-making should be conducted openly and discussed adequately with full regard to ethical considerations and not cost alone [15].

In Trinidad & Tobago, healthcare is provided free for the citizens at the public hospitals and there is a need for healthcare providers to look into potential cost-saving measures, particularly in the ICU, which consumes a significant amount of hospital resources. With this background, this study was designed to evaluate the total resources consumed by the non-survivors during their ICU stay and the costs associated with it. In addition, the study also sought to determine if there were potential factors to indicate that ICU care could have been non-beneficial in some non-survivors and if the costs in such patients could have been minimized. The study was intended to provide data on the resource consumption of ICU non-survivors so as to help in decision-making pertinent to ICU admission of moribund patients. The other benefits may include the possible application of the concept of medical futility and early withdrawal of care decisions by the physicians and the family members, which may also assist in ICU quality improvement.

## Materials and methods

Approval was obtained from the Ethics Committee of The University of the West Indies, St. Augustine, Trinidad & Tobago. Since this was a retrospective chart review, a waiver of individual patient consent was approved. Patient confidentiality was maintained by not including patients’ names or other identifiers during data collection process and by assigning codes to patients.

Inclusion criteria were patients of age eighteen years or older, who died in adult ICU after 24 hours of admission when there was availability of complete clinical notes and daily ICU flowsheets.

Exclusion criteria were patients of age seventeen years or under, patients who died in the ICU within 24 hours of admission, and patients whose complete file and all the data required for the study were unavailable.

Setting

In the hospital where the study was conducted, adult patients are admitted to the six-bedded ICU admitting patients from the emergency department, medical and surgical wards, and the operating theatres (post-operative surgical patients). The ICU is managed by anesthesiologists in conjunction with primary admitting teams, and the nurse to patient ratio is 1:1. The medical staff including one consultant, one registrar, and two house-officers are available round the clock. The ICU has state-of-the-art equipment with a wide range of invasive monitoring including intra-arterial and central venous lines, portable radiograph, ultrasonography, and other therapeutic interventions such as continuous renal replacement therapy (CRRT), intra-aortic balloon counterpulsation (IABP), and hemodialysis. The unit however does not have an extracorporeal membrane oxygenator (ECMO). Patient records are maintained and updated manually, and there are no existing electronic medical records and computerized database in the setting.

Study design and data collection

This was designed as an initial chart-review, followed by a prospective cost estimation for non-survivors in the adult ICU over a period of nine months during 2016. Any patient who was admitted to ICU for more than 24 hours and did not survive to discharge from the ICU was considered as a ‘non-survivor’ for the purpose of the study. The complete medical files (including the daily ICU charts) of these patients were obtained from the medical records department. Data were collected from the daily patient notes and ICU flowsheets, which included patient age, gender, admission diagnosis, length of ICU stay (LOS), number of days of mechanical ventilation, organ systems affected, and physiological variables for the simplified acute physiology score - version II (SAPS II) at admission, from which predicted mortality rate was calculated.

In order to estimate costs by the ‘bottom-up’ approach, the following data were collected for each patient:

1. Number of arterial blood gas samples (ABGs) done within the ICU

2. Number and type of radiological investigations (Plain X-ray, CT scan, etc.,)

3. Number and type of laboratory tests (hematology, biochemistry, microbiology, etc.,)

4. Daily consumption of all the drugs (including inotropes, sedatives, antibiotics, colloids including human albumin, blood products)

5. Daily consumption of consumables including syringes, gloves, catheters, cannulae, dressing, enteral feeds, gases for mechanical ventilation, consumables associated with monitoring, etc.,

6. Special interventions including continuous renal replacement therapy (CRRT), intra-aortic balloon pump (IABP), total parenteral nutrition (TPN), etc.

The costs for the various therapeutic interventions, drugs, laboratory investigations, radiological investigations, blood products, special therapeutic supports (CRRT, IABP, TPN, etc.,) were calculated based on the number of ‘units’ of such items consumed by each patient in the study as documented in the daily progress notes and flow sheets and the cost of each unit is obtained from the respective departments like purchasing, administration & finance, etc., The drug costs were obtained from the pharmacy supervisor, sourced from the pharmaceutical price list prescribed by the Ministry of Health, Trinidad & Tobago.

The cost of blood products such as packed cells, platelets, and fresh frozen plasma was obtained from the senior technician at National Blood Transfusion Centre, Port of Spain, Trinidad as a closest estimate correlating to the price of packed red cells used in one study of intra-operative transfusion management in a cardiac surgery unit in Trinidad & Tobago in 2009 [16].

Laboratory and radiology investigation costs were obtained from the National Costing of Health Services in Trinidad & Tobago by the Health Economics Unit, The University of the West Indies.

Daily patient clinical support services and consumables syringes, lines, circuits, tubing, etc., and intensive nursing care, which are common to all patients in ICU were obtained as nursing activity score, which is a modified therapeutic intervention scoring System (TISS-28) score [17]. The nursing activity score for each patient per day and for the total length of stay were calculated.

The costs for medical staff were derived by apportioning the average number of hours spent by each grade of staff (consultant, registrar, and house officer) respectively per patient per day based on the calculated hourly salaries.

The statistical package for social sciences (SPSS) (version-23) for Windows was used for data analysis. Statistical significance was fixed at the level of p<0.05.

## Results

**Figure 1:**
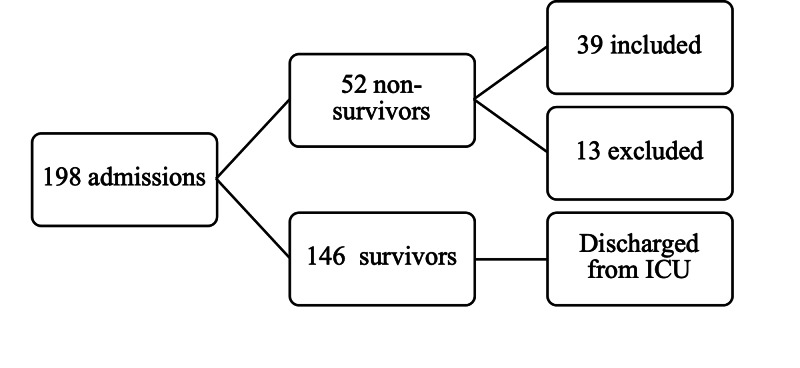
Flowchart of selection of patients

This is a convenient sample pragmatic study enrolling patients during the time period selected for the research. A total of 198 patients were admitted to adult ICU during nine months of 2016. Fifty-two (52) patients died in ICU during the study period, but only thirty-nine (39) patients met the inclusion criteria. The following flowchart (Figure 1) describes the patients included in the study:

The age of the patients ranged from 19 to 83 years, with a median age of 56 years [Interquartile range, IQR (45, 66)]. The length of stay (LOS) had a wide range of one to 27 days, with a median of five days (IQR 2, 10). The median SAPS II score was 62 (IQR 52, 73). The median predicted mortality rate was 71.9 (IQR 50.7, 87.1). The median number of organs failed was 3.0 (IQR 2, 3). The median days of mechanical ventilation were five days (IQR 2, 10). Gender distribution was comparable, 21 patients (53.8%) were male and 18 (46.2%) were female. On admission, 33.3% were diagnosed with cardiovascular problems, 28.2% were surgical, 10.3% had respiratory illnesses, 10.3% neurological illnesses and 17.9% had other illnesses including sepsis, renal disorders, poly-trauma, and poisoning among others. Based on the patient’s specific diagnosis, ischaemic heart disease was the most common diagnosis (17.9%), followed by pneumonia (10.3%). The average score for nursing workload was found to be 28 points.

The severity of illness depicted by SAPS II score, when analyzed based on the specific organ system involved, showed that patients diagnosed with cardiovascular system (CVS) illnesses to be having the highest mean SAPS II score of 72.3 [Standard Error (SE) 6.3], followed by neurology 64.2 (SE 6.5), respiratory 56.7 (SE 13.3), while surgical cases had the least score of 9.5 (SE 5.9).

The mean length of stay (LOS) was the highest for the patients with respiratory problems (11.25, SE 3.3) days, followed by surgical (10.6, SE 2.8), neurology (5.2, SE 2.4), and cardiovascular ailments (3.8, SE 0.7).

The total cost of providing ICU care for the non-survivors during the study period was US$ 765,232, from which an average cost of providing ICU care per patient was derived at US$ 19,621.

**Figure 2:**
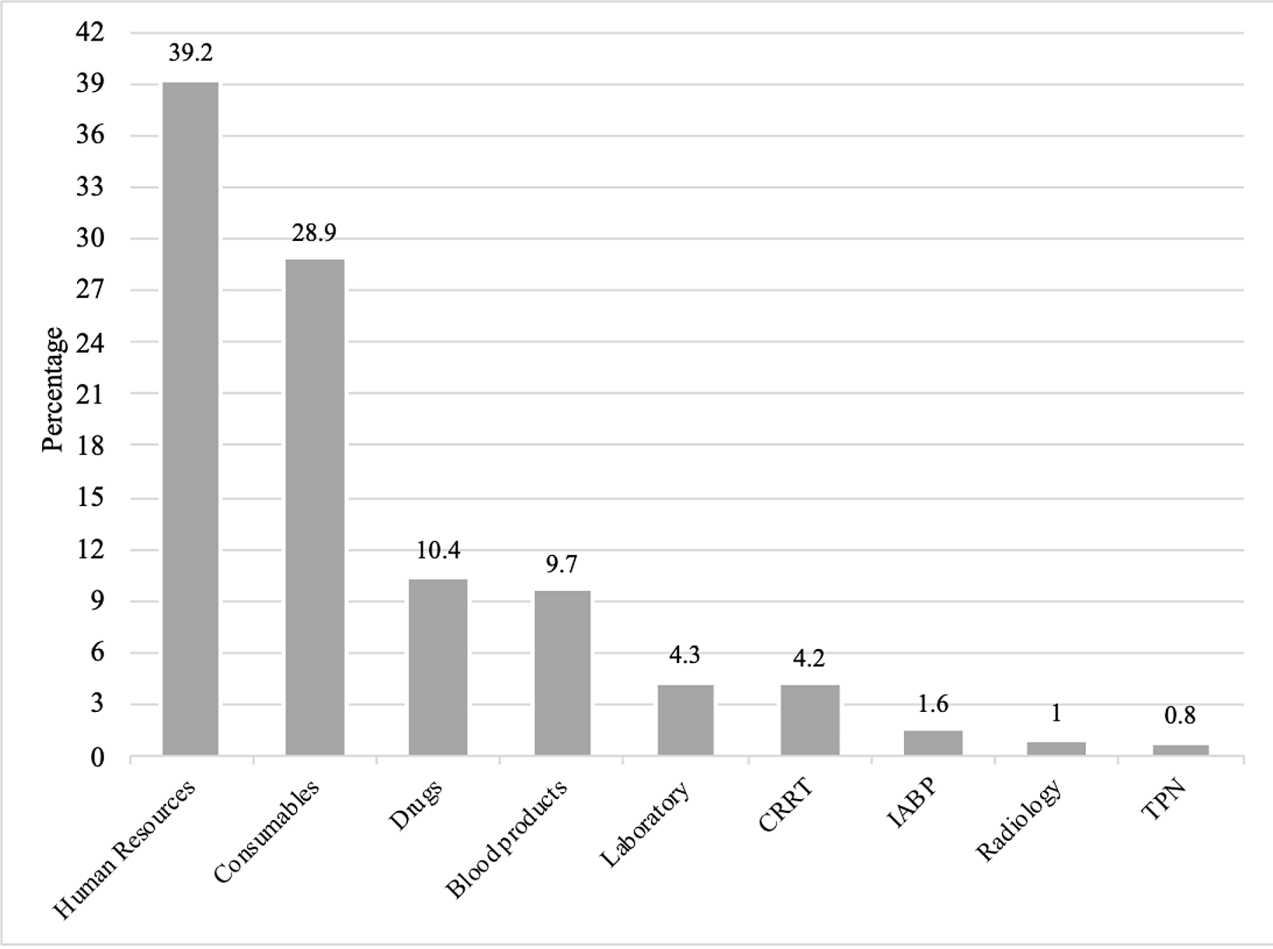
Distribution of the costs for individual components CRRT = Continuous Renal Replacement Therapy IABP = Intra-aortic Balloon Pump TPN = Total Parenteral Nutrition

According to the breakdown of costs, staff costs accounted for the highest being US$ 299,903, followed by intervention costs (as quantified by nursing activity including consumables, enteral feeds, etc.) US$ 221,200; drugs amounted to US$ 79,445, blood products US$ 74,062, laboratory investigations US$ 32,732, and radiology investigations US$ 12,096. Of the total patients studied, eight received continuous renal replacement therapy (CRRT) with a cost of US$ 31,875, two received IABP costing US$ 7812 and two others received total parenteral nutrition (TPN) with a cost of US$ 6300. Figure 2 depicts the proportion of individual components of the costs.

Table 1 compares the total costs expended between groups of patients, elderly (≥65) versus younger patients, patients who had a SAPS II score ≥62 (median value) versus those who had less than 62, and also between those who had a cardiac arrest before admission to ICU versus those who did not have an arrest.

**Table 1:** Comparison of different variables and costs SAPS II: Simplified Acute Physiology Score - version II

Variable		Number	Total costs (US$)	Mean costs (US$)
Age (y)	≥65	10	155,524.69	15,552
	<65	29	609,708.12	21,024
SAPS II score	≥62	18	492,109.53	27,339
	<62	21	273,123.28	13,005
Cardiac arrest	YES	3	72,017.03	24,005
	NO	36	693,215.78	19,256

Of all the patients, twenty-nine (29) patients were of age <65 years and the average cost for providing ICU care for this group was US$ 21,024, while there were 10 patients who were in the elderly category, the average costs being US$ 15,552. Similarly, there were 18 patients who had a high SAPS II score of ≥62, and the average cost was US$ 27,339, while the average cost for 21 patients who had a lower SAPS II score (<62) was US$ 13,005.

The average cost for patients who did not suffer a cardiac arrest before admission was US$ 19,256, which was lesser when compared to the cost for patients who suffered a cardiac arrest, which amounted to US$ 24,005. Non-parametric analysis by Mann-Whitney test did not yield any statistically significant difference between the above groups, although the trend of expenditure was higher in patients with higher SAPS II scores and patients who suffered a cardiac arrest before ICU admission.

Table 2 shows the total costs expended in accordance with the system affected.

**Table 2:** Total costs in different diagnostic groups * Sepsis, renal disorders, poisoning, etc.

System affected	Total Costs (US$)
Cardiovascular system (n=13)	63854.41
Respiratory system (n=4)	147873.28
Neurological system (n=4)	75214.05
Postoperative surgical patients (n=11)	164576.22
Others* (n=7)	178568.81

The higher the SAPS II score, the lower was the LOS (length of stay) [Pearson correlation (2-tailed) p= 0.01]. Also, the total costs were less for patients with higher SAPS II scores (mostly because their LOS was less) [Pearson correlation (2-tailed), p= 0.03].

## Discussion

Costs for providing intensive care are significantly high in any hospital system and the overall patient outcomes in each unit depend on various factors including the demographics of the population group, disease severity, clinical practices, and the organization of the healthcare system. The present study focused on estimating the costs for providing care specifically in non-survivors because this group of patients would have presumably consumed much more resources in the ICU due to the increased severity of illness; however, did not survive despite receiving full critical care support with multiple interventions.

The overall mortality rate in the ICU during the study period was 26.2%, which is comparable to published data from other countries, ICU mortality in the USA during 2014 was 10-29% [3].

The median age of the non-survivors was 56, which is relatively younger given the fact that ICU utilization by the geriatric age group is consistently increasing in recent decades [18]. This may be explained by the fact that postoperative surgical patients and miscellaneous categories formed a major proportion of the patients enrolled in the study.

In a similar vein, the relatively higher proportion of postoperative surgical patients within non-survivors also must be explained. These patients underwent major surgeries including thoracic aortic aneurysm, neurosurgery for brain tumors, and Whipple’s procedure for pancreatic cancers, and got admitted to the study ICU during the study period. As expected, their SAPS II scores were also relatively lower.

The present study showed that non-survivors consumed a significant amount of resources during a nine-month study period amounting to more than three-quarters of a million US dollars. If this is extrapolated to annual expenditure, it amounts to almost 30% of the total allocated annual budget for the study hospital, which was approximately US$ 3.86 million during 2015.

The average cost of providing care to a non-survivor in the present study was US$ 19,621 per patient. A study conducted in the USA during the same time period of the current study, reported a mean cost per ICU stay to be US$ 16,353 [18]. In comparison, more than a decade before, another study conducted in Barbados, another Caribbean country showed an average cost per patient for the non-survivors to be US$ 10,445 in 2002 [9].

The high costs for non-survivors may be attributable to the severity of illness at the time of admission to ICU, which is obvious from the high SAPS II scores in the first 24 hours of ICU admission. The mean SAPS II score for the non-survivors in the present study was 64.7. This may imply that the patients have a higher tendency to consume a wide range of resources and therapeutic interventions during the first few days of their ICU stay, contributing to the increase in the total costs. The length of stay also determined the total costs incurred. This finding is comparable to an earlier report from the USA, wherein there was a 12% increase in the overall costs in patients who did not survive the ICU, who also had an extended ICU stay [18].

However, it is interesting to note that the patients with very high SAPS II scores had a shorter length of stay, probably because of their moribund severity of illness and early death. As a corollary, the total costs incurred for the patients with very high SAPS II scores were also less, which can be explained by the shorter length of stay. This may be an important point to consider when ICU admission policies have to be guided by the severity of illness scoring systems. Whether it may be possible to implement ICU gatekeeping strategies and triage the patients before ICU admission, or to withdraw treatment at an earlier date in those patients with high predicted mortality, the appropriate threshold for these scores should be considered for the purpose of prognostication and discussion among the caregivers and the family members.

Shortage of beds in ICU is a common scenario worldwide and bed allocation is considered as one of the most difficult and stressful aspects of work in ICU physicians [19]. Being aware that the ICU beds and the attendant expensive resources are limited, physicians often struggle with decision-making when there is a need to admit the patient, and the clinical status is compounded by the constraints of cost. A lot of effort has been put into defining the admission criteria for ICUs and formulating the requisite guidelines. Patients who clinically present too sick to be benefitted by any ICU intervention, should not be admitted to the ICU for aggressive care [20].

However, these decisions are extremely hard to make for any critical care team, particularly in our ICU settings in Trinidad, where healthcare is provided free of cost to citizens. Our ICU occupancy rate has been always above 80% implying that ICUs are always full at any given time and patients requiring ICU will be managed at the emergency oom and general wards. As a corollary, there will be always a staff shortage in out ICUs. Earlier studies from other regions have found varying prevalence of this refusal to ICU admission ranging from 24 to 38% [21, 22]. A large proportion of patients who were not admitted to ICU had more severe illness as reflected by their high APACHE II scores [22]. However, relying on a scoring system alone for decision-making regarding ICU admission is highly controversial due to the inherent pitfalls of prognostic models.

With respect to the breakdown of costs, the proportion of costs incurred for staff forms the majority in the present study (>60%), which is a common finding in most cost estimation studies from different parts of the world [23-27]. As mentioned earlier, since the ICU occupancy rates are always high, there were no staff awaiting patient admission and contributing to the 'sunk' costs. However, when cost-cutting measures are considered, this component must be taken into consideration as to how to minimize staff costs. In recent times, many ICUs have started telemedicine to partly address this issue, although there can be other unique problems associated with this measure [28, 29]. 

Cost-saving measures can be safely implemented in the ICUs without any potential effect on the patient outcomes, e.g., reducing the use of human albumin, choosing a cheaper antibiotic in place of an expensive one, cutting down the number of X-rays done routinely in the ICUs, etc. The newer tools developed by the Institute for Healthcare Improvement (IHI) for ‘waste’ identification in ICU care should be utilized to measure the prevalence and the causes for inappropriate use of ICU beds and to identify the most common causes of waste in ICUs such as delays in procedures, delays in the transfer of patients out of ICU and end-of-life decision-making [30].

The paradigm of futile care in ICU, otherwise termed as inappropriate care, exists in most ICUs worldwide. But, even acknowledging its existence and the ongoing inappropriate care is a frustrating topic for most critical care clinicians, due to the lack of clear guidelines and policies governing it. There have been attempts to formulate recommendations and guidelines by Committees established by various international critical care societies [10]. None of the Caribbean countries have statutory support for Advanced Directives and Living Wills and hence these options are ruled out. There is a need for public engagement efforts and advocacy for policies and legislation regarding the appropriate use of life-prolonging technologies and withholding them when there are clear indications that the care will be non-beneficial. The decision to withdraw or withhold aggressive care is being considered by the doctors and nurses without policy and statutory support, and most of the time they end up with frustration and decreased job satisfaction, emotional and psychological burnout. Since futility decisions are taken predominantly by the healthcare workers and not at the request of patients and/or surrogates, much more education is needed for healthcare providers regarding non-beneficial care.

There are some limitations to the present study which include a smaller sample size, non-inclusion of survivors for comparison, and the possible minor inaccuracies in the data provided by the various departments for cost estimation. However, most cost estimation studies are fraught with similar issues [6]. In order to minimize limitations, a single researcher (VG) collected all the data to ensure uniformity and obtaining data from sources without changing the definitions.

## Conclusions

In conclusion, the present study quantified the costs for providing ICU care for non-survivors and the individual components of these costs. Overall, the costs were found to be high despite the fact that the patients did not survive the ICU stay. Strict ICU admission policies and guidelines should be formulated to reduce costs in critical care incorporating the severity of illness scoring systems such as the SAPS II score and other clinical criteria. Non-aggressive palliative care integrated into ICU care has been recommended by the Society of Critical Care Medicine, USA (IPAL-ICU). These guidelines also may be used to avoid wastage of costs, by reducing unnecessary investigations, unnecessary drugs including antimicrobials guided by the antibiogram, once the clinicians decide that the patient is moribundly ill. Although inappropriate care in ICU and end-of-life palliative care are difficult issues to deal with, the recommendations from the leading critical care societies around the world should guide clinicians in appropriate and timely decision-making, which might possibly reduce non-beneficial care in ICU and thereby reducing wastage of resources.
